# *Xiphopterella devolii* (Polypodiaceae), a new species and newly recorded genus in Taiwan

**DOI:** 10.1186/1999-3110-54-24

**Published:** 2013-08-30

**Authors:** Shann-Jye Moore, Barbara S Parris, Tzu-Tong Kao, Pi-Fong Lu, Wen-Liang Chiou

**Affiliations:** 1 Taiwan Society of Plant Systematics, 88, Ting-Chow Rd., Sec 4, Taipei, 11677 Taiwan; 2 Fern Research Foundation, 21 James Kemp Place, Kerikeri, Bay of Island 0230, New Zealand; 3 Taiwan Forestry Research Institute, 53 Nan Hai Road, Taipei, 10066 Taiwan

**Keywords:** Grammitid fern, Polypodiaceae, Spore, Taiwan, Taxonomy, *Xiphopterella devolii*

## Abstract

**Background:**

Grammitid ferns are a tropical monophyletic clade nested in Polypodiaceae, containing more than 20 genera and more than 750 species. Many of them also grow in Taiwan. During the survey of recent two decades, an unknown grammitid fern was discovered and the taxonomic treatment is given herein.

**Results:**

A new species, collected from Taiwan, is recognized and named, i.e., *Xiphopterella devolii* S. J. Moore, Parris, & W. L. Chiou. The holotype is deposited in TAIF, and isotypes are in HAST, K, L, US, and TNS. It is also distributed on SE & S China. The genus *Xiphopterella* is also a new record to Taiwan.

**Conclusion:**

A new species, *Xiphopterella devolii* S. J. Moore, Parris, & W. L. Chiou is documented herein. The *Xiphopterella* is a new recorded genus in Taiwan and is first found beyond Malesia regions.

**Electronic supplementary material:**

The online version of this article (doi:10.1186/1999-3110-54-24) contains supplementary material, which is available to authorized users.

## Background

Grammitid ferns are a tropical monophyletic clade nested in Polypodiaceae (Schneider et al., 2004; Schuettpelz and Pryer, 2007; Sundue et al., 2010), containing more than 20 genera and more than 750 species (Parris, 2007). In Taiwan, six genera/17 species (DeVol, 1975), four genera/18 species (Kuo, 1985), six genera/19 species (Shieh et al., 1994; Lu and Yang, 2005), 6 genera/18 species (Yang and Liu, 2002), or 3 genera/21 species (Knapp, 2011) have been documented. Here we report a new species, *Xiphopterella devolii*, which is also a newly recorded genus, in Taiwan.

## Methods

### Morphological study

Observations and measurements of trichomes and spores were based on specimens cited herein under different kinds of microscopes.

### Spores

Mature spores of *Xiphopterella devolii* were collected from opened sporangia on the type specimen (*SJ Moore 24567*) and observed with a Leitz DMR light microscope under differential interference contrast (DIC). Diameters of 100 spores were measured. To increase the field depth of presented images, a series of photos were pictured under bright field (BF) with 2 μm focus intervals and merged by Helicon Focus 4.03 (Helicon Soft). Spore topology was observed with a scanning electron microscope (SEM). The spores were spread on a cover glass coated with Stay-on adhesive (Surgipath), coated with gold by IB-2 ion coater (Eiko Engineering), and observed with a TM3000 tabletop SEM (Hitachi). The backscattered electron (BE) images were photographed under 15 kV accelerating voltage.

### Trichomes

Scales and hairs were also observed with a light microscope and SEM. To avoid damaging the type specimen (*SJ Moore 24567*), the whole specimens were observed under low vacuum mode of the SEM without pretreatment (i.e., fixation, dehydration, and coating).

## Results

### Taxonomic treatment

**Xiphopterella** Parris, Gard. Bull. Sing. 58(2): 249. 2007.

Plants small, epiphytic. Rhizomes radial, with stipes in whorls; scales not clathrate, pale reddish brown, glabrous. Stipe not articulate, phyllopodia absent. Lamina pinnately divided; lateral veins 1-forked when fertile, free, each vein ending with a hydathode on adaxial surface. Hairs simple and 1- to 3-forked with eglandular branches. Sori superficial. Sporangia glabrous.

About seven species in the world. They are mainly distributed on Malesia regions, especially Peninsular Malaysia (Parris, 2007). The discovery of this genus beyond Malesia regions is first documented here.

**Figure 1:**
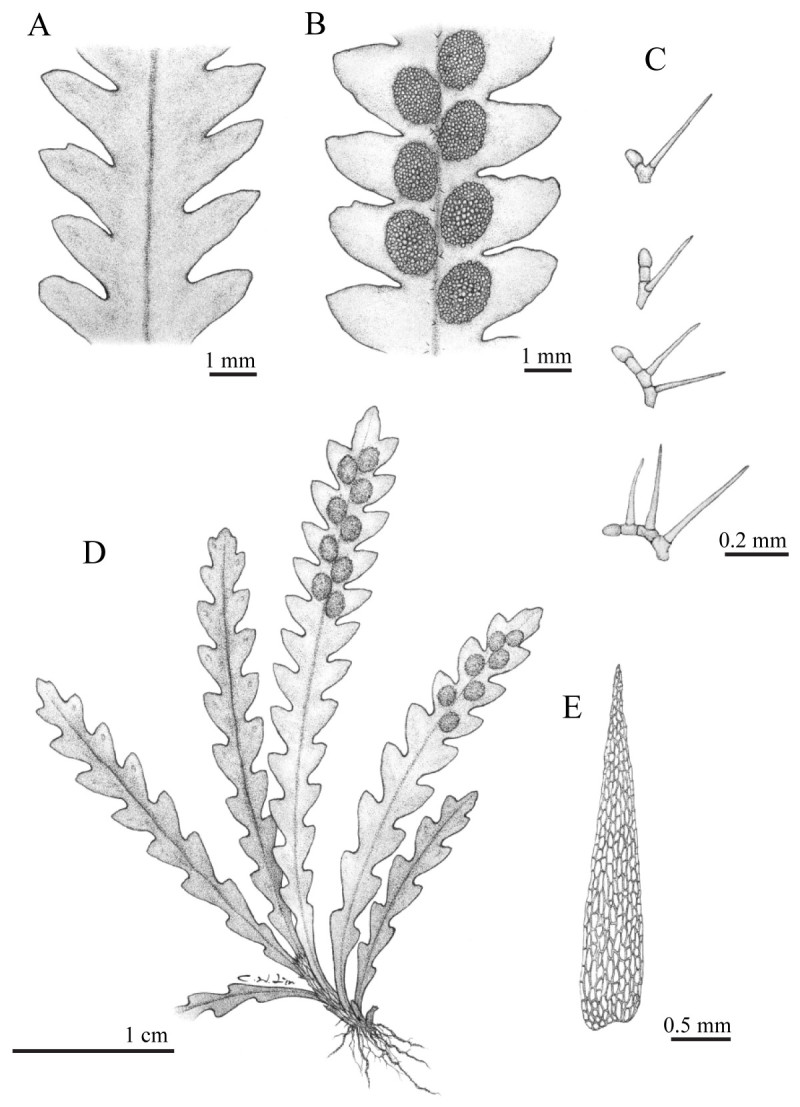
***Xiphopterella devolii*** **S. J. Moore, Parris** **&** **W. L. Chiou. A**. Adaxial view of portion of frond. **B**. Abaxial view of portion of frond. **C**. Simple septate and forked hairs with non-septate branch(es). **D**. Habit. **E**. Rhizome scale.

**Figure 2:**
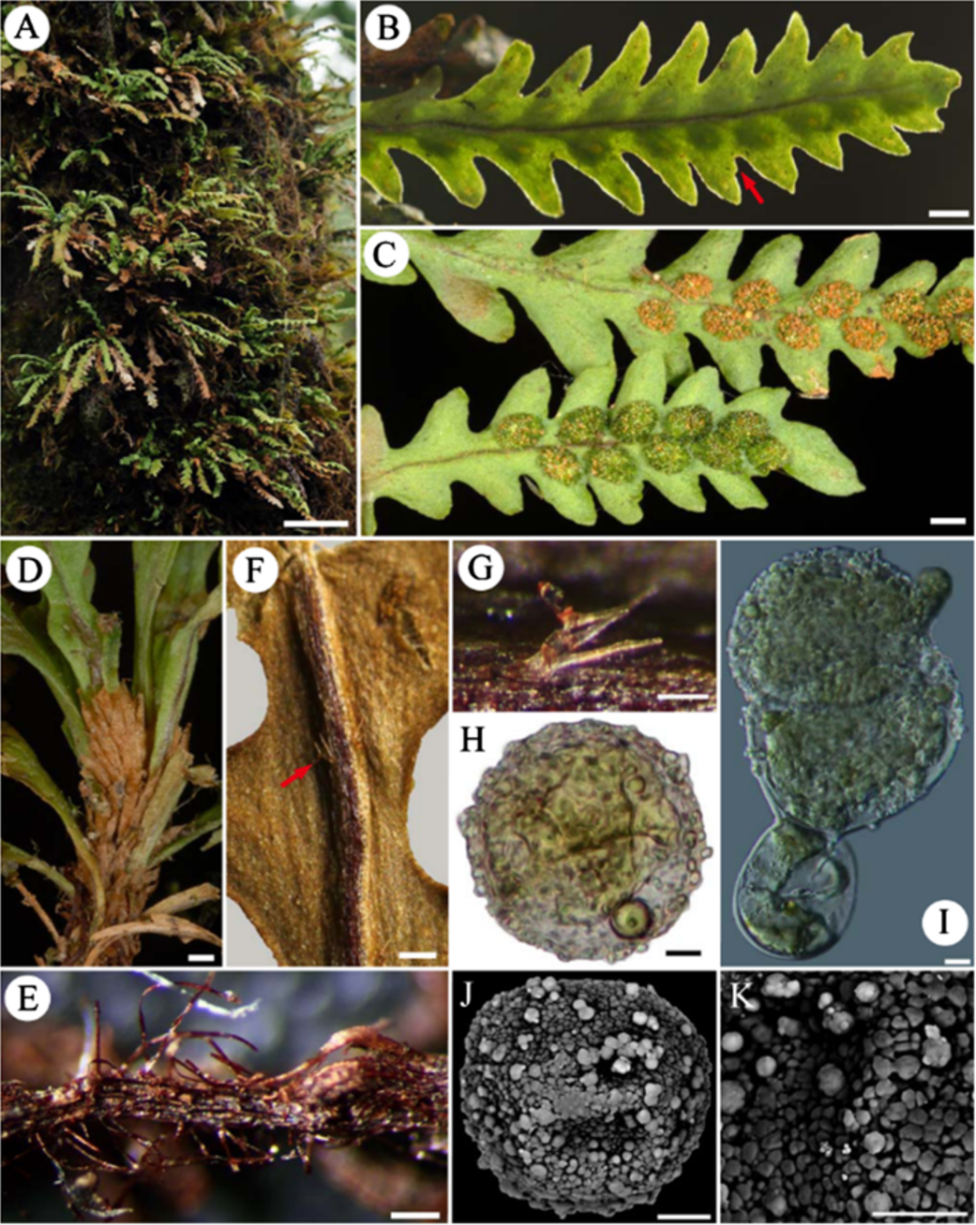
***Xiphopterella devolii*** **S. J. Moore, Parris** **&** **W. L. Chiou. A**. Habit and habitat (bar = 30 mm). Small epiphytes growing on tree trunks in dense moist forest. **B**. Adaxial view of fertile frond (bar = 1 mm). Lateral vein forked, ended with a hydathode; segment margin entire, rarely with a blunt tooth at acroscopic margin (arrow). **C**. Abaxial view of fertile frond (bar = 1 mm). Lower: mature sori with green spores. Upper: spore-released sori. **D**. Lower portion of plant showing brown scales on rhizomes (bar = 0.5 mm). **E**. Portion of root with reddish hairs (bar = 0.2 mm). **F**. Portion of lamina showing forked hairs (arrow) on abaxial midrib (bar = 0.5 mm). **G**. Simple septate and 3-forked hair with non-septate branches (bar = 0.1 mm). H-K. Spores (bar = 5 μm). **H**. Light microscopic image (BF). **I**. Germinated spore found on specimen (DIC). **J**. SEM image (BE). **K**. Portion of spore surface. Papillate with sparse globules.

**Xiphopterella devolii** S. J. Moore, Parris & W. L. Chiou, sp. nov.-TYPE: TAIWAN. Ilan County, Sunglo Lake, 20 July 2000, *SJ Moore 24567* (holotype: TAIF; isotype: HAST, K, L, US, TNS) Figures 1 and 2.

Plants epiphytic. Roots filamentous wiry, ca. 0.2 mm thick; hairs simple, shiny to brick red, 0.2-0.5 mm long. Rhizomes radial; scales pale brown to brown, ovate to lanceolate, 1–2.5 mm long, ca. 0.5 mm wide, not clathrate, entire, glabrous. Stipes sessile or nearly so. Laminae linear, linear-elliptic, or linear-oblanceolate, 2–7 × 0.4-0.9 cm, acute at apex, attenuate to form a wing at base, simple, pinnatifid; pinnatifid segment inclined or ascending, widely to narrowly triangular, slightly oblique or falcate, up to 5 mm, entire, or rarely with a small blunt tooth at acroscopic margin; rachis prominent on abaxial side, grooved on adaxial side; lateral veins hidden, invisible, even by transmitted light, tip with a hydathode, simple in sterile segment, forked in fertile segment, acroscopic branch not extending beyond sorus; hairs transparent to pale, mainly on abaxial side of rachis and at base of laminae of young fronds, simple septate and 1 to 2 (or 3) forked with non-septate branches, the simple septate branch 0.1-0.2 mm long, 2–4 cells with an apical reddish club-like head, the lateral branch 0.2-0.3 mm long. Sori round to oval. Spores green, globose to tetrahedral-globose, 24.58 ± 0.49 μm in diameter, usually depressed, some germinate in sporangium, surface papillate with sparse globules.

### Additional specimens examined

**TAIWAN**. I-Lan, Sunglo Lake, 13 May 1999, *C. C. Chen 7260* (TAIF); same locality, 20 May 2006, *T. C. Hsu 509* (TAIF); same locality, 30 July 2006, *P. F. Lu 12177* (TAIF); same locality, 23 Aug 2009, *P. F. Lu 18727_1* (TAIF).

### Distribution and ecology

Taiwan and SE & S China. In Taiwan, it grows on tree trunks in dense moist forest; 1250–1350 m a.s.l.

### Etymology

This new species is dedicated to Charles E. DeVol (1903–1989), a kind taxonomist who contributed to the research of Taiwanese lycophytes and ferns very much and was one of the editors for the Flora of Taiwan, 1st ed.

## Discussion

This new species was first discovered almost 15 years ago when it was suspected to be *Grammitis cornigera* (Baker) Ching or even a new species of *Xiphopteris*(Chen 1998). The species *Ctenopterella cornigera* (Baker) Parris (syn. *Micropolypodium cornigerum* (Baker) X. C. Zhang) is endemic to Sri Lanka. Many Chinese specimens (eg., *PE 24845*, *PE 1366173*, *PE 02114785*, *PE 02185396*) identified as *Micropolypodium cornigerum* (or *Xiphopteris cornigera* (Baker) Copel. and *Grammitis cornigera* (Baker) Ching) are in fact this new species. The ‘*Grammitis* sp.’ in Knapp (2011) is attributed to this new species, too.

## Conclusion

Through detail comparison with previous literatures and specimens in worldwide herbaria, a grammitid fern is confirmed to be a new species, i.e., *Xiphopterella devolii* S. J. Moore, Parris, & W. L. Chiou. Its types are designed and located herein. The *Xiphopterella* is a new recorded genus in Taiwan and is first found beyond Malesia regions.

## Authors’ original submitted files for images

Below are the links to the authors’ original submitted files for images. Authors’ original file for figure 1 Authors’ original file for figure 2
