# Supplementary figures and images for: Xiphopterella devolii (Polypodiaceae), a new species and newly recorded genus in Taiwan
Source: Bot Stud. 2013 Aug 30;54:24. doi: 10.1186/1999-3110-54-24 (PMC5432846; doi:10.1186/1999-3110-54-24)

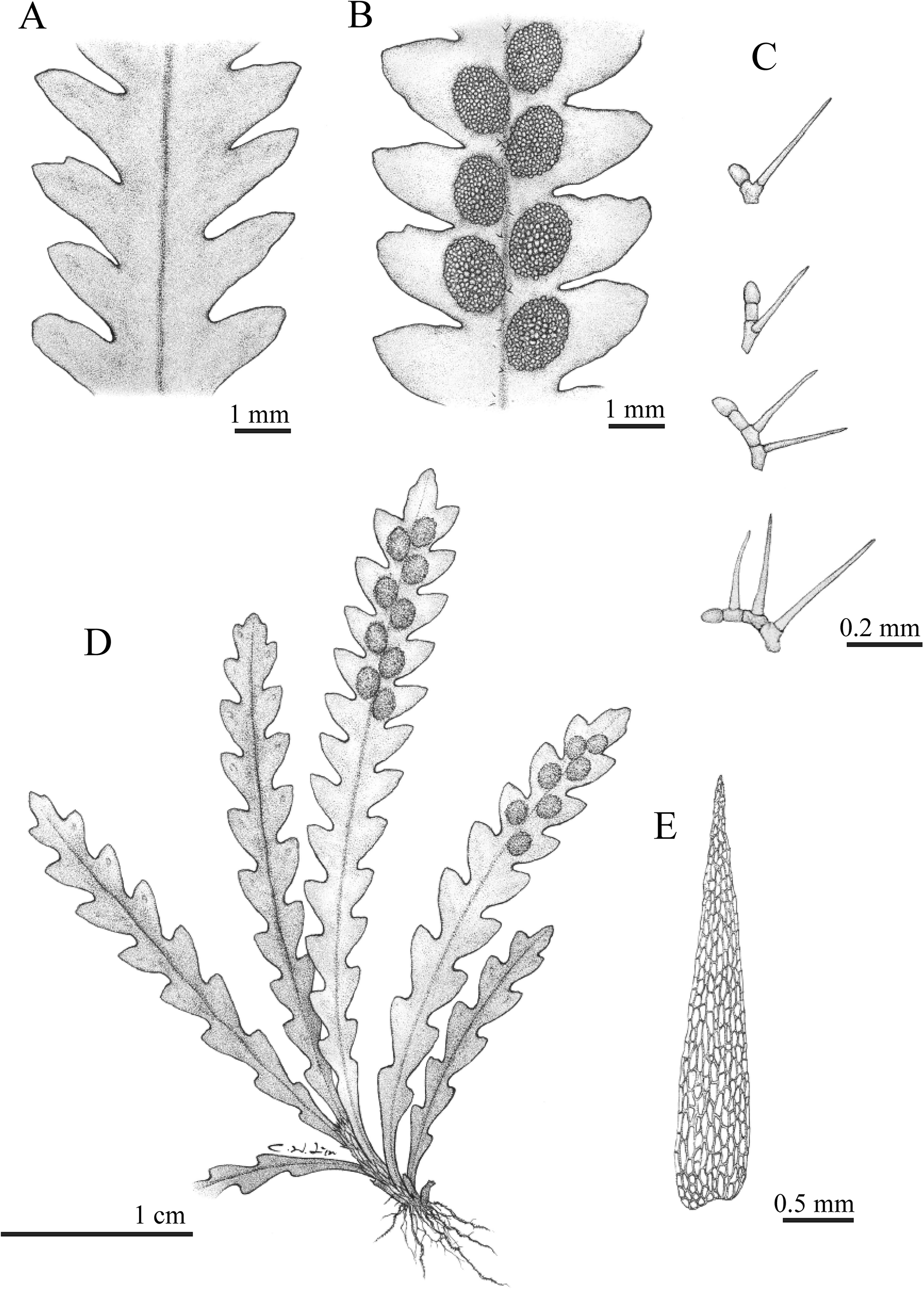

Supplement: Supplementary file 1 — Authors’ original file for figure 1 [file 40529_2012_20_MOESM1_ESM.tif]

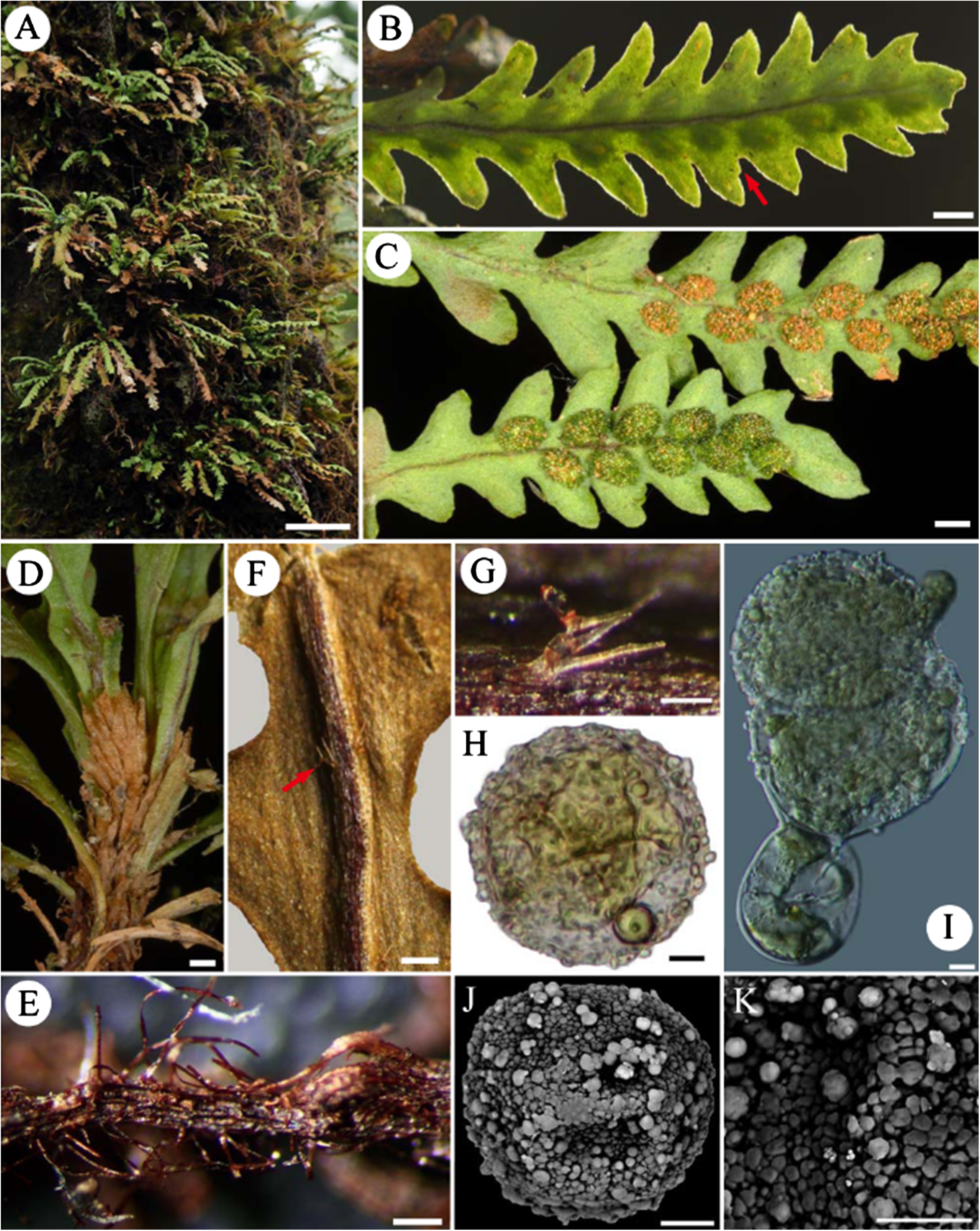

Supplement: Supplementary file 2 — Authors’ original file for figure 2 [file 40529_2012_20_MOESM2_ESM.tif]
